# Evaluation of right myocardial performance index of in vitro fertilization fetuses and spontaneous pregnancy fetuses: a cross-sectional study

**DOI:** 10.1186/s12947-021-00242-5

**Published:** 2021-01-29

**Authors:** Shaoqi Chen, Zemin Zhuang, Qingzi Chen, Xiya Du, Weiping Li, Xuerui Tan

**Affiliations:** 1 Department of Ultrasound, First Affiliated Hospital of Shantou University Medical College, Shantou, 515041 China; 2 The Clinical Research Center of the First Affiliated Hospital of Shantou University Medical College, Shantou, 515041 China

**Keywords:** Right myocardial performance index (RMPI), In vitro fertilization (IVF) fetuses, Spontaneous pregnancy (SP) fetuses, Normal reference range

## Abstract

**Background:**

Whether the in vitro fertilization (IVF) has an effect on the cardiac function of the fetus is very important to evaluate the safety of the technique. The aim of this paper is to establish normal reference range for the fetal right myocardial performance index (RMPI), and compare the reference range between IVF fetuses and spontaneous pregnancy (SP) fetuses by automatic measurement of the RMPI.

**Methods:**

Three hundred seventy-one spontaneous singleton pregnancies (the control group) and 39 singleton pregnancies conceived by IVF (the experimental group) were enrolled into the current study. An automatic measurement system was used to acquire the RMPI. The cardiac function of the two groups was compared by t-test.

**Results:**

There was no significant difference in normal reference range of RMPI between IVF fetuses and SP fetuses (RMPI 0.42 ± 0.05 vs 0.43 ± 0.05). No strong correlation was also noted between RMPI with gestational age and heart rate.

**Conclusions:**

Normal reference ranges of RMPI of IVF fetuses and SP fetuses were established, and no significant difference between IVF fetuses and SP fetuses in RMPI was found. Thus, these findings may suggest that IVF has little impact on cardiac function of the fetus.

**Supplementary Information:**

The online version contains supplementary material available at 10.1186/s12947-021-00242-5.

## Introduction

Assisted reproductive technology (ART) was developed to address many causes of infertility, providing many people hope of becoming parents. ART includes procedures, such as in vitro fertilization (IVF), intracytoplasmic sperm injection (ICSI), cryopreservation of gametes or embryos, and/or the use of fertility medication. However, its safety is still controversial, and the birth defects of its offspring have greatly attracted scholars’ attention. Yan et al. reported that there was no significant increase in birth defects in the ART offspring [1]. However, a number of scholars pointed out that infants born after ART treatment had a higher risk of birth defects than those who conceived spontaneously, and the risk of congenital heart defect (CHD) in ART fetuses was higher than that in spontaneous pregnancy (SP) fetuses [2, 3]. Children conceived by ART manifested the existence of cardiac and vascular remodeling in utero life and persisted in postnatal life, suggesting opportunities for early detection and potential intervention [4]. However, several pregnant women prefer IVF in ART, thus it is of great importance to compare the safety of IVF with ICSI.

The myocardial performance index (MPI) was first proposed by Tei et al. [5] for the evaluation of heart function in adults with dilated cardiomyopathy. The non-invasive, Doppler-derived MPI has been reported to be useful as a combined index of global myocardial function. The MPI is defined as the sum of isovolumetric contraction time (ICT) and isovolumetric relaxation time (IRT) divided by ejection time (ET) [6–8]. However, due to anatomical factors, numerous scholars have concentrated on the left MPI (LMPI). To our knowledge, the right ventricle indicates physiological dominance and is more sensitive to deviation in the fetus, thus, the right ventricle may change before the left when neonatal hypoxia-ischemia happens. In other words, the right MPI (RMPI) may be more representative and can sensitively reflect the fetal cardiac function.

Although the MPI is a promising indicator, the measurement of traditional RMPI is more complicated compared with the LMPI. Recently, automatic measurement of fetal right MPI (RMPI) from pulsed wave Doppler spectrum was described by Suresh et al. [9]. This method can simplify the measurement of RMPI. In the present study, we aimed to validate a new measuring tool to measure the RMPI of SP fetuses and IVF fetuses, and compare the reference range of IVF and SP fetuses.

## Methods

### Study population

This was a prospective cohort study approved by the Ethics Committee of the First Affiliated Hospital of Shantou University Medical College (Shantou, China). Written informed consent was obtained from all participants. From September 2019 to January 2020, 410 participants, who were at 19–40 weeks’ gestation, were enrolled into the current study. There were 371 spontaneous singleton pregnancies (average age 29.35 ± 4.37 years) that were matched as control group and 39 singleton pregnancies conceived by IVF (average age 32.03 ± 4.12 years). Patients were excluded if they had any maternal diseases, such as high blood pressure and diabetes. Additionally, if there were any fetal malformations, e.g. CHD at the time of undergoing ultrasound or later diagnosis of any fetal malformation, the patients were excluded as well.

### Echocardiogram and cardiac Doppler

**Fig. 1:**
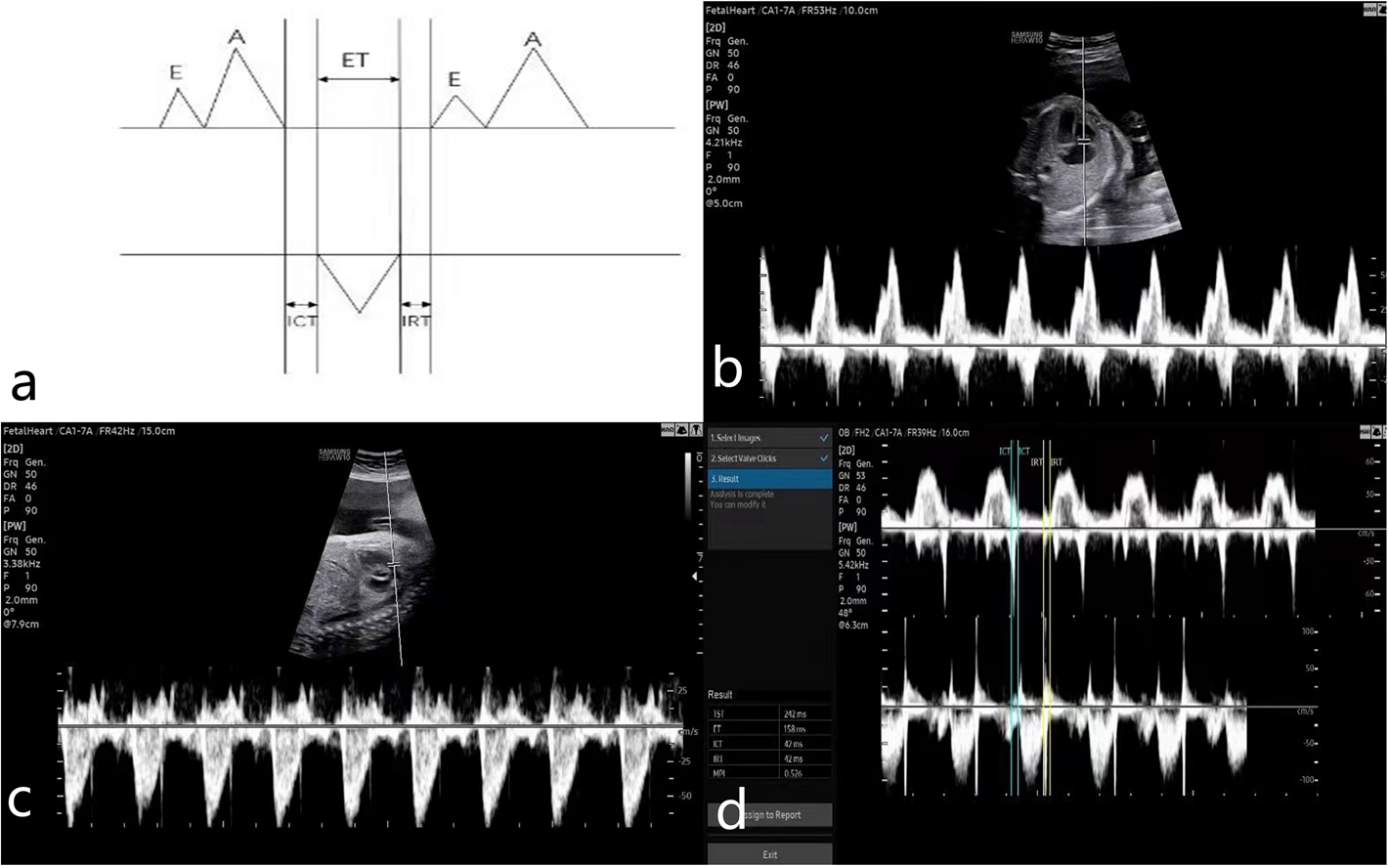
The measurement of right myocardial performance index (RMPI). **a** A simple Model of RMPI Measurement. **b** The inflow image of the tricuspid leaflets. **c** The outflow image of the pulmonary leaflets. **d** The image of RMPI measurement

Transabdominal fetal ultrasound was performed to obtain images for analysis, employing an HERA W10 ultrasound system (Samsung, Seoul, Korea) equipped with a 1–7 MHz probe. Mechanical and thermal indices were defined < 1.0 and < 2.0, respectively. The angle of insonation was kept below 30°. Routine examination and measurement of fetus, including biparietal diameter (BPD), head circumference (HC), abdominal circumference (AC), foot length (FL) and hand length (HL). The measurement of RMPI included two steps. First, the transverse four-chamber view of the fetal thorax with an apical or bottom heart and the outflow tract of right ventricle were obtained to show the tricuspid valve and pulmonary valve clearly, and the Doppler gate was put at the tips of the tricuspid and pulmonary leaflets to capture the inflow images and the outflow images, respectively. The images showed opening and closing of the valves. Second, the inflow images and the outflow images were captured together (Fig. 1). Lastly, an automatic measurement system applied a novel “coarse-to-fine” strategy to detect the coarse cardiac cycle (CC) and valve click (VC) range (inflow/outflow peaks) [9]. The system can give right myocardial performance Index (RMPI), total spent time (TST), isovolumetric contraction time (ICT), isovolumetric relaxation time (IRT) and ejection time (ET) automatically. Three times readings were obtained and averaged per examination.

The sampling volume was placed under the orifice of the tricuspid valve from the apical four-chamber view or the bottom four-chamber view to acquire the ratio of E wave/ A wave peak velocities (TV-E/A) of tricuspid valve. It was measured for three times in a row, and the average value was taken.

### Statistical analysis

SPSS 23.0 software (IBM, Armonk, NY, USA) was used to perform statistical analysis. Kolmogorov-Smirnov test (K-S test) and Shapiro-Wilk test (S-W test) were applied to assess normal distribution of the data. Normally distributed data were expressed as mean values ± standard deviation (SD). Student’s t-test was utilized for comparing normally distributed data, including RMPI, TST, ICT, IRT, ET and TV-E/A between IVF fetus and SP fetus. The relationships of the RMPI with gestational age (GA) and heart rate (HR) were analyzed by linear regression analysis. *P*-value< 0.05 were considered as statistically significance.

## Results

### Participant characteristics

The general data of pregnant women and fetuses in the two groups were shown in Table 1. The values of age and heart rate (HR) of IVF group were higher than that of the SP group. The data of age and HR showed statistically significant differences (*P* < 0.05). However, There were no statistical differences in gestational age (GA), biparietal diameter (BPD), head circumference (HC), abdominal circumference (AC), foot length (FL) and hand length (HL) between the two groups (*P* > 0.05).

**Table 1:** The general data of the study population

Variable	IVF group (***n*** = 39)	SP group (***n*** = 371)	*P*-value
**Age (years)**	32.03 ± 4.12	29.35 ± 4.37	0.000*
HR (bpm)	145.75 ± 9.15	149.10 ± 9.16	0.030*
GA (weeks)	29.59 ± 4.73	30.13 ± 5.41	0.549
BPD (mm)	74.28 ± 13.78	75.32 ± 13.90	0.658
HC (mm)	270.95 ± 44.10	273.64 ± 47.33	0.734
AC (mm)	254.97 ± 49.97	259.65 ± 56.49	0.620
FL (mm)	55.90 ± 10.40	56.84 ± 12.25	0.642
HL (mm)	50.10 ± 8.34	50.69 ± 9.88	0.721

*GA* Gestational age, *HR* Heart rate, *BPD* Biparietal diameter, *HC* Head circumference, *AC* Abdominal circumference, *FL* Foot length, *HL* Hand length

*P < 0.05, significantly different from the control group

### Fetal cardiac function analysis

**Fig. 2:**
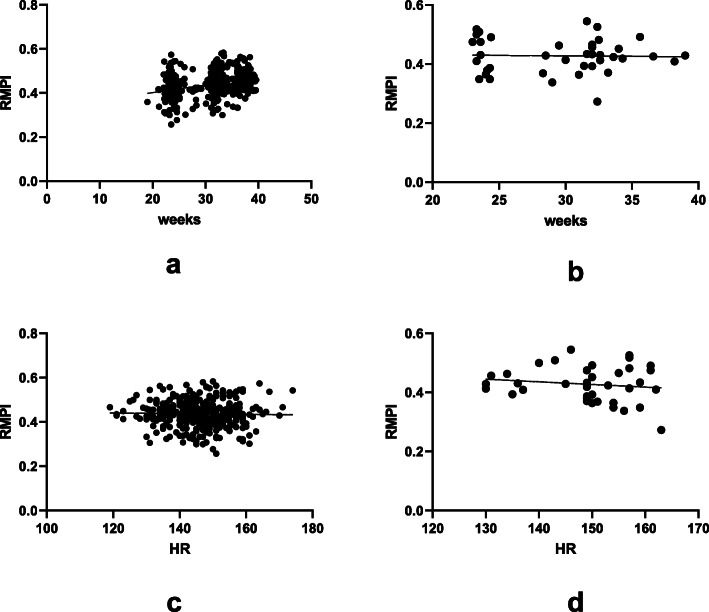
Linear regression analysis between right myocardial performance index (RMPI) and gestational age (GA). **a** Correlation between RMPI and GA in SP fetus (*r*^*2*^ = 0.1015). **b** Correlation between RMPI and GA in IVF fetus (*r*^*2*^ = 0.0007). **c** Correlation between RMPI and HR in SP fetus (*r*^*2*^ = 0.0005). **d** Correlation between RMPI and HR in IVF fetus (*r*^*2*^ = 0.0196)

The comparison of fetal cardiac function between the two groups was summarized in Table 2. There were no statistical differences in TST, ICT, IRT, ET, RMPI and TV-E/A between IVF fetuses and SP fetuses (*P* > 0.05). The value of RMPI of IVF fetuses and SP fetuses is 0.42 ± 0.05 vs 0.43 ± 0.05. In correlation analysis (Fig. 2), there was no strong correlation between RMPI and GA, and between RMPI and HR in SP fetuses and IVF fetuses.

**Table 2:** Fetal cardiac function parameters

Variable	IVF fetuses (***n*** = 39)	SP fetuses (***n*** = 371)	*P*-value
TST (ms)	242.92 ± 13.73	248.06 ± 15.64	0.053
ICT (ms)	28.36 ± 10.27	30.98 ± 9.99	0.120
IRT (ms)	44.08 ± 10.12	44.23 ± 10.02	0.926
ET (ms)	169.97 ± 10.12	172.86 ± 11.43	0.133
RMPI	0.42 ± 0.05	0.43 ± 0.05	0.891
TV-E/A	0.75 ± 0.22	0.74 ± 0.15	0.611

*TST* Total spent time, *ICT* Isovolumetric contraction time, *IRT* Isovolumetric relaxation time, *ET* Ejection time, *RMPI* Right myocardial performance index, *TV-E/A* Ratios of E wave/ A wave peak velocities (TV-E/A) of tricuspid valve

## Discussion

There are increasingly IVF fetuses, thus, it is very beneficial for patients to indicate whether the fetal cardiac function is abnormal or not by non-invasive methods. The ejection fraction (EF) and ratios of E wave/A wave peak velocities (E/A) commonly used in clinic are only the evaluation of systolic function or diastolic function. However, the systolic and diastolic function influence each other when the blood flow index of fetal heart changes, so it is more accurate to evaluate the overall function of fetal heart. Therefore, it is highly essential to determine the reference range of RMPI.

Although the age and HR in the IVF group were higher than those in the SP group, there was no significant difference in RMPI between the two groups, which reflected that the age and HR had no significant effect on RMPI. In this study, linear regression analysis was used to show that there was no strong correlation between RMPI and GA, and between RMPI and HR in SP fetuses and IVF fetuses (Fig. 2). Ghawi et al. reported that MPI is independent from GA and HR [10]. They pointed out that systolic ventricular function changes from high- to low-level, while diastolic ventricular function is gradually matured from low- to high-level with the increase of GA [10]. MPI is advantageous for comprehensive evaluation of cardiac systolic and diastolic ventricular functions, therefore, this may be the reason why MPI does not change with GA.

The method of automatic measurement of RMPI used in the present study is time-saving, eliminating the need to measure ICT and IRT time, respectively. This is also essential to measure the spectrum of tricuspid valve and pulmonary valve, put the two pictures together, and then select the appropriate cardiac cycle (CC), and the system will automatically calculate the values of ICT, IRT, ET, and MPI. The values measured by this method are similar to those reported by Ghawi et al. [10] and Hamela-Olkowska et al. [11].

The present study unveiled that there was no significant difference in normal reference range of RMPI between IVF fetuses and SP fetuses. This may suggest that there was no obvious cardiac abnormality in IVF fetuses during the fetal period, or the change of cardiac function may not occur before the measuring moment of the IVF fetus. The normal reference range of cardiac RMPI in the SP fetus and the IVF fetus can be shared as well.

Valenzuela-Alcaraz et al. pointed out that the cardiac and vascular remodeling exists in the fetuses received by ART [4]. In theory, compared with IVF fetuses, ICSI fetuses were not fertilized by natural selection. During the operation of ICSI, there is a greater threat to children’s health. Compared with IVF, ICSI bypasses the natural selection barrier of oocytes to sperm and may inject poor quality sperm to pass on certain genetic defects and high genetic risk genes to the next generation [12, 13]. This may indicate that ART technology may affect fetal cardiac function, while IVF may be relatively safe. However, we still cannot conclude that IVF has no influence on fetal cardiac function.

### Limitations

There were a number of limitations in the present study. Firstly, the sample size of IVF fetuses was extremely small. Secondly, we did not collect ICSI fetuses for the study. Collecting ICSI fetuses for making comparison with other parameters may increase the accuracy of our findings. In addition, this was a cross-sectional study and was limited to the second and third trimesters of pregnancy [14]. Last but not the least, our study lacked an invasive gold standard to determine the accuracy of RMPI measurements. Thus, further experiments with large sample size are required to confirm our findings and eliminate the above-mentioned deficiencies.

## Conclusions

During fetal period, no significant difference was noted in RMPI between IVF fetuses and SP fetuses. This may suggest that IVF is more secure than ART technology. Therefore, the normal range of RMPI values of SP fetuses and IVF fetuses can be shared during fetal period.

## Supplementary Information

**Additional file 1.**

## Data Availability

The data and materials used in this study are available from the corresponding author on reasonable request.

## Abbreviations

IVF: In vitro fertilization
SP: Spontaneous pregnancy
RMPI: Right myocardial performance index
LMPI: Left myocardial performance index
SD: Standard deviation
ICT: Isovolumetric contraction time
IRT: Isovolumetric relaxation time
ET: Ejection time
TST: Total spent time
ART: Assisted reproductive technology
ICSI: Intracytoplasmic sperm injection
CC: Cardiac cycle
VC: Valve click
CHD: Congenital heart defect
HR: Heart rate
GA: Gestational age
BPD: Biparietal diameter
HC: Head circumference
AC: Abdominal circumference
FL: Foot length
HL: Hand length
TV-E/A: Ratio of E wave/ A wave peak velocities of tricuspid valve
EF: Ejection fraction
